# Effect of subacromial erosion shape on rotator cuff and clinical outcomes after hook plate fixation in type 5 acromioclavicular joint dislocations: a retrospective cohort study

**DOI:** 10.1186/s12891-021-04987-y

**Published:** 2022-01-10

**Authors:** Ho-Seok Oh, Sungmin Kim, Jeong-Hun Hyun, Myung-Sun Kim

**Affiliations:** Department of Orthopedic Surgery, Chonnam National University Hospital, 42 Jebong-ro, Dong-gu, Gwangju, 61469 South Korea

**Keywords:** Acromioclavicular joint dislocation, Clavicle, Coracoclavicular distance, Hook plate fixation, Rotator cuff lesion, Shoulder, Subacromial erosion

## Abstract

**Background:**

Surgical fixation using hook plates is widely used in the treatment of acromioclavicular (AC) joint dislocations. The purpose of this study was to evaluate the incidence and shape of subacromial erosions after removal of the hook plate in type 5 AC joint dislocations. Further, we evaluated the effect of the shape of the subacromial erosion on the rotator cuff.

**Methods:**

We retrospectively reviewed 30 patients who underwent hook plate fixation for type 5 AC joint dislocations at our hospital between December 2010 and December 2018. Patients with a follow-up of at least 1 year were included. Clinical outcomes were assessed using the final follow-up Constant-Murley, Korean Shoulder, and visual analog scores. To ensure that the appropriate reduction was well maintained, the coracoclavicular distances of the injured and contralateral sides were evaluated at the last follow-up. Computed tomography was performed to investigate the presence and shape of the subacromial erosion after hook plate removal at 4 months after surgery. Ultrasonography was performed to investigate the presence of rotator cuff lesions at the last follow-up. Clinical and radiological outcomes were compared between groups divided according to the presence and types of subacromial erosions.

**Results:**

Subacromial erosion was observed in 60% of patients (18/30): 13, 2, and 3 simple groove, cave, and marginal protrusion types, respectively. Four patients showed reduction loss at the final follow-up. There were no significant differences in clinical and radiological outcomes between the groups with and without subacromial erosion. Moreover, there were no significant differences between groups according to the types of subacromial erosion. There were no rotator cuff lesions, such as partial tears, in the injured shoulders.

**Conclusions:**

Hook plate fixation may induce subacromial erosions. However, the subacromial erosions caused by the hook plate did not affect the clinical outcomes of type 5 AC joint dislocations. Moreover, regardless of its shape, the subacromial erosion did not affect the clinical outcomes nor cause rotator cuff lesions after plate removal.

## Background

Acromioclavicular (AC) joint dislocation is a common traumatic injury in the upper extremities, accounting for approximately 9% of all shoulder injuries. Moreover, up to 43.5% of athletes have AC joint dislocation after direct trauma to the shoulder [11, 16, 17, 22, 26, 28]. AC joint dislocations are classified into six types based on the extent of displacement of the clavicle relative to the acromion [1, 11, 21, 25]. For AC joint dislocation types 4–6, surgical indications are essential. For type 3 dislocations, the decision on conservative and surgical treatment is controversial [3, 8, 20, 23, 29].

Various surgical options are available for the treatment of acute AC joint dislocations. These include bandaging, fixation of the AC joint with pins, tension band wiring using the modified Weaver-Dunn procedure, fixation with washer and screw, and clavicular plate [5, 7, 13, 27]. Among these, the hook plate fixation technique shows excellent security. Its minimal surface contact yields sufficient blood supply and permits horizontal stability as a concomitant result of subacromial fixation [17, 29].

Stable fixation and early return to mobilization of the affected part are the main advantages of using a hook plate. However, previous studies have reported complications such as shoulder stiffness, subacromial erosion, impingement, and rotator cuff tear [4, 10, 12, 30]. The hook plate can be rigidly fixed on the clavicle while remaining mobile beneath the acromion. This may induce a pressure rise in the hook under the surface of the acromion causing erosion [30]. Some studies have evaluated the contact characteristics between the acromion and hook plate. These studies concluded that the pinpoint between the hook plate tip and the under surface of the acromion is the main factor inducing subacromial erosion [14, 30].

Many studies have revealed that the shape of the acromion affects the pathology of the rotator cuff. Thus, surgeons traditionally perform acromioplasty at the time of rotator cuff repair [2]. Outcomes after acromioplasty in the treatment of rotator cuff disease were good when surgeons converted a “curved” or “hooked” acromion into a “flat” shape [2, 9, 18]. Likewise, we hypothesized that if the subacromial erosion has a protruding shape so-called type III in our study, impingement of the rotator cuff can cause a rotator cuff lesion. To the best of our knowledge, there is lack of studies whether the subacromial erosion shape affects clinical outcomes and complications, such as shoulder impingement and rotator cuff lesions, in patients who undergo hook plate fixation.

This study analyzed clinical and radiological results after hook plate fixation surgery in patients with AC joint dislocation. The aim was to evaluate the incidence and shape of subacromial erosions after removal of the hook plate in type 5 AC joint dislocations. Further, the study evaluated the effect of the shape of the subacromial erosion on the rotator cuff.

## Methods

We enrolled 30 patients (26 men and 4 women; mean age, 47.5 years) who underwent hook plate fixation for AC joint dislocations at our hospital between December 2010 and December 2018. Cases with a follow-up period of at least 1 year were retrospectively analyzed. We only included patients with Rockwood classification type 5 AC joint dislocations [22] for consistency in evaluation conditions. Patients with clavicular fracture, contralateral upper arm impairment, nerve injury, previous surgical history in the same shoulder, and shoulder dysfunction due to previous injury were excluded. The mean follow-up duration was 31.0 months (range, 12.0–49.2 months). The hook plate was removed after a mean time interval of 4.6 months (range, 3.0–8.5 months).

The study was approved by the ethics committee of the institutional review board (IRB) of Chonnam National University Hospital (CNUH-2020-383). All methods were performed in accordance with the relevant guidelines and regulations. Written informed consent to participate was obtained from all the patients.

### Surgical technique

**Fig. 1:**
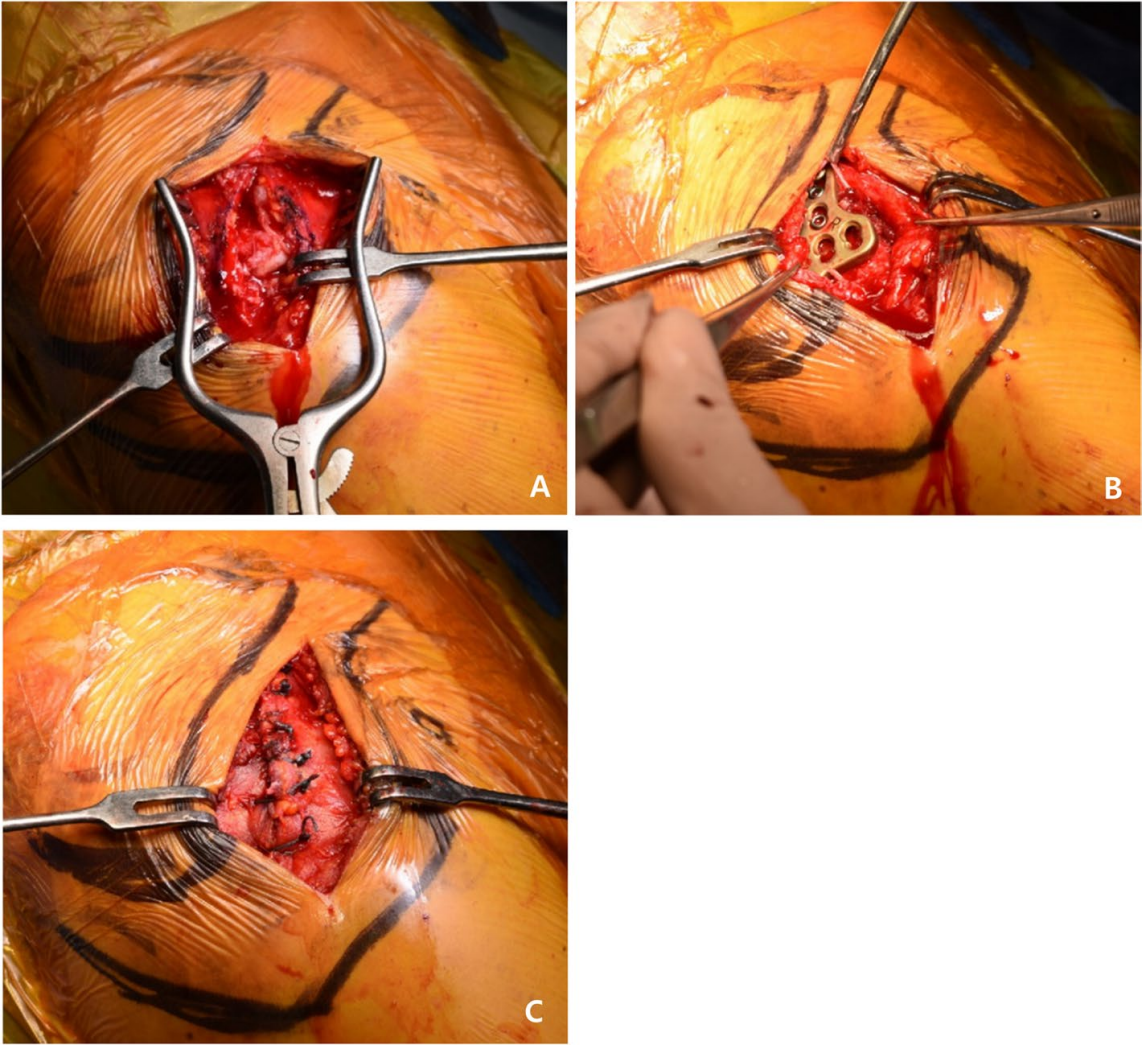
**A** The AC joint is exposed and reduced by direct visualization and fluoroscopy. **B** An LCP clavicle hook plate is inserted into the rear bottom of the acromion and proximal end of the plate. **C** Deltoid and trapezius muscle fascia are sutured at the avulsion site. AC, acromioclavicular; LCP, locking compression plate

All surgeries were performed by a single orthopedic surgeon (MSK). Under general anesthesia, the patient was placed in the beach chair position. An anterior approach to the lateral part of the clavicle was used to perform a 4 cm straight incision. After cutting the deltotrapezial fascia, the joint was reduced by direct visualization and fluoroscopy (Fig. 1A). An appropriately sized locking compression plate clavicle hook plate (DePuy Synthes, Oberdorf, Switzerland) was bended to fit the contour of acromion and clavicle, inserted into the rear bottom of the acromion, and the proximal end of the plate was fixed to the clavicle using several screws (Fig. 1B). Subsequently, the deltoid and trapezius muscle fascia were sutured at the avulsion site (Fig. 1C).

### Postoperative rehabilitation

After surgery, an arm sling was applied for 1 to 3 weeks depending on the patient’s pain to protect the shoulder. Passive shoulder mobilization was initiated as soon as the postoperative pain had decreased. All patients were allowed to use their arms for daily activities. Non-restricted movement and strength-related activities were allowed 6 weeks and 3 months, respectively, after the surgery.

### Radiological evaluation

**Fig. 2:**
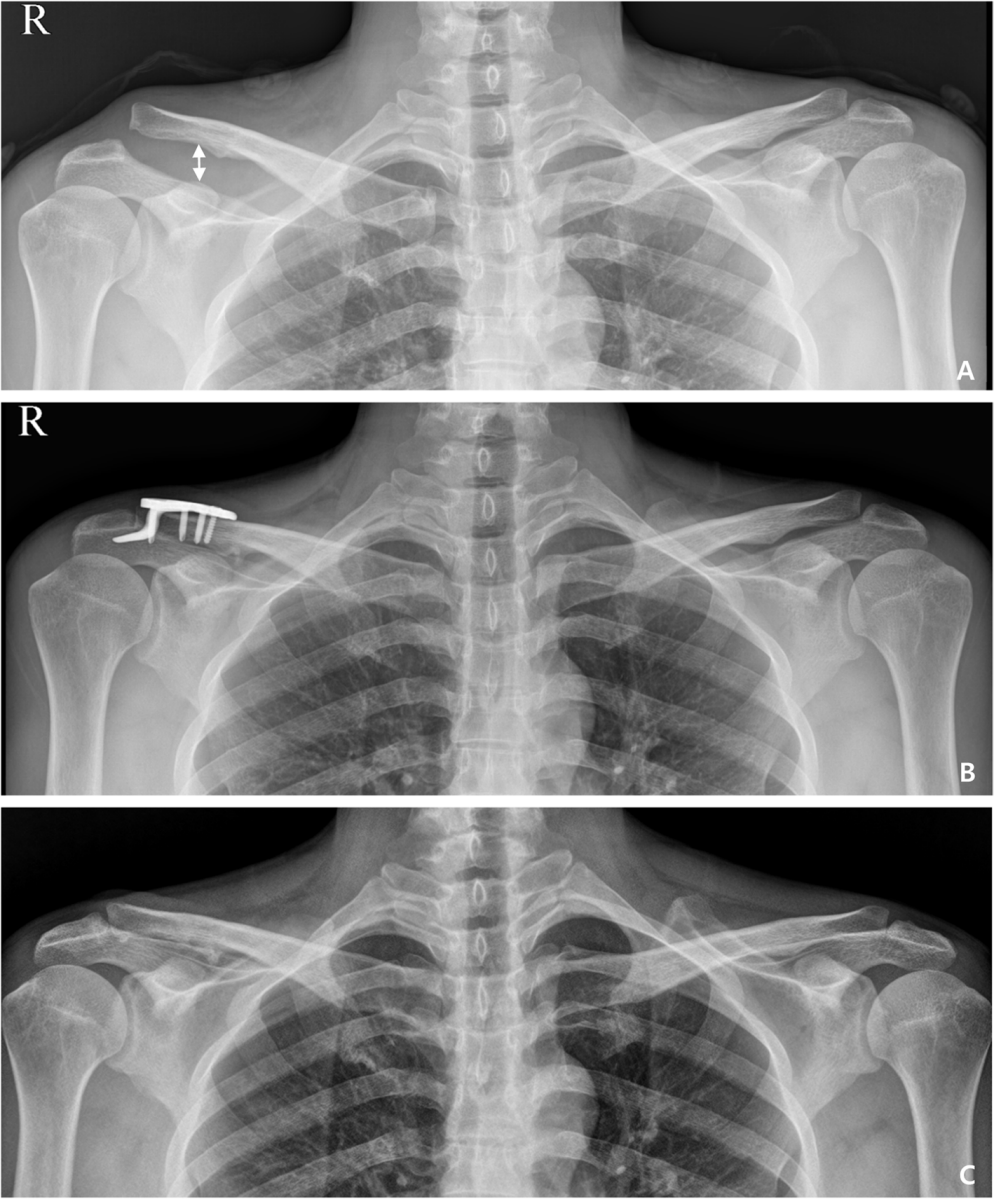
A 34-year-old male patient treated with hook plate fixation for acromioclavicular joint dislocation: (**A**) Preoperative (white arrow: coracoclavicular distance), (**B**) postoperative, and (**C**) last follow-up after implant removal

**Fig. 3:**
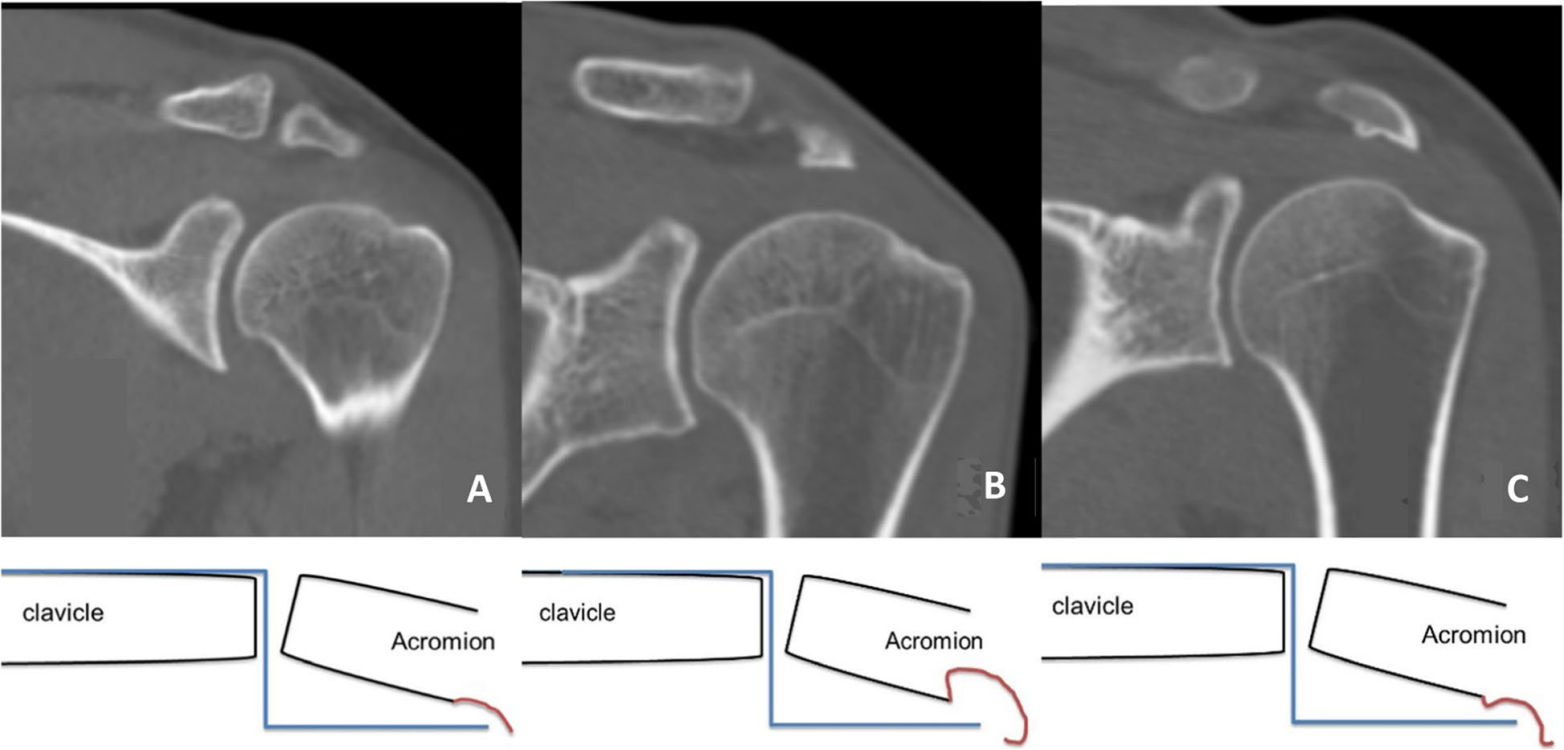
Computed tomography is used to decide acromial erosion type (**A**: Type I - simple groove, **B**: Type II - cave, **C**: Type III - marginal protrusion)

The anterior-posterior (AP) view of both shoulders was taken to evaluate the alignment of the lateral clavicle with the acromion on every follow-up day (Fig. 2). In all the patients, subacromial erosion was evaluated by computed tomography (CT) after implant removal. The type of subacromial erosion was determined based on the cut where the shape of the subacromial erosion was most clearly visible in the CT coronal view. When the width of the erosion at the upper level is the same or narrower than the width of the erosion at the acromial undersurface, the erosion is defined as type I (shallow groove). When the width of the erosion at the upper level is wide, it is defined as type II (cave). Bone protrusion around the subacromial erosion was defined as type III (marginal protrusion) (Fig. 3). We compared the clinical and radiological outcomes by dividing the patients into groups with and without subacromial erosion. Further, the patients were divided into three groups according to the subacromial erosion type (simple groove, cave, and marginal protrusion).

The coracoclavicular distance (CCD) of the injured and contralateral sides was evaluated by measuring the vertical distance between the upper border of the coracoid process and the lower end of the clavicle. This was done in the AP view of both shoulders at the final follow-up (Fig. 2). We considered subluxation when the CCD increased from 50 to 100%. Dislocation was considered when the CCD was over 100% with respect to the contralateral side as assessed on final follow-up radiographs [10]. At the last follow-up, to compare CCDs, patients were divided into two groups according to the presence or absence of subacromial erosions. The CCDs were also compared in three groups divided according to subacromial erosion type. Additionally, we evaluated rotator cuff lesions using ultrasonography performed by a single orthopedic surgeon (MSK) at before surgery and the final follow-up.

All radiographic measurements were accurately assessed using a picture archiving and communication system (PACS; Maroview version 5.4; Marotech Inc.). Two orthopedic surgeons who were blinded to the surgical treatment performed the assessment.

### Clinical evaluation

Clinical function was evaluated using the Constant-Murley score (CMS), Korean Shoulder score (KSS), range of motion (ROM), and visual analog scale (VAS) score. For the CMS, subjective and objective clinical outcomes were included with a maximum score of 100 points: pain, 15 points; activities of daily living, 20 points; ROM of the shoulder, 40 points; and muscle power, 25 points [6]. The KSS includes five domains totaling 100 points: function, 30 points; pain, 20 points; satisfaction, 10 points; ROM, 20 points; and muscle power, consisting of strength, 10 points; and endurance, 10 points [24]. The VAS was used to measure pain, with 0 indicating no pain and 10 indicating extremely severe pain. Measurements were performed at the final follow-up by an independent observer who was not involved in the study. An orthopedic surgeon who was not involved in the study measured the ROM using a full-circle manual goniometer.

### Statistical analyses

Data were analyzed using SPSS Statistics for Windows, version 18.0 (SPSS Inc., Chicago, Ill., USA). A paired *t*-test was used to determine the significance of intergroup differences in clinical and radiological results between groups divided by the presence or absence of a subacromial erosion. Kruskal Wallis test was used to determine the significance of intergroup differences in clinical and radiological results between groups divided by subacromial erosion type. A *P*-value less than 0.05 was considered statistically significant.

## Results

### Clinical and radiological outcomes

Of the total (*n* = 30) patients, 18 showed subacromial erosion and 12 showed no subacromial erosion. Subacromial erosions were divided into three types according to shape: 13, 2, and 3 patients had subacromial erosions of types I, II, and III respectively.

Both groups showed significantly good clinical results. There were no significant differences in clinical results, including ROM, between the patients with and without subacromial erosions. Mean CCDs were also not significantly different at the final follow-up in patients with and without subacromial erosions. Further, there were no significant differences in the period from surgery to removal in both groups for patients with and without subacromial erosions (Table 1).

**Table 1:** Clinical and radiological outcomes compared between patients with and without subacromial erosion at final follow-up

	Subacromial erosion (***n*** = 18)	No subacromial erosion (***n*** = 12)	***P***-value
Removal time	4.61 ± 0.94	4.48 ± 1.33	0.771
Functional score			
CMS	96.72 ± 4.81	94.50 ± 5.52	0.252
KSS	97.06 ± 5.43	94.17 ± 5.18	0.157
VAS	0.94 ± 0.83	0.94 ± 0.58	0.717
Range of motion			
Forward elevation	154.44 ± 21.75	159.17 ± 14.43	0.515
Abduction	155.56 ± 21.75	153.33 ± 30.85	0.818
External rotation at side	63.33 ± 18.15	59.17 ± 7.93	0.462
CC distance (%)	26.05 ± 45.84	33.85 ± 48.23	0.658

*CMS* Constant-Murley score, *KSS* Korean Shoulder score, *VAS* visual analog score, *CC* coracoclavicular

When the patients were divided into groups according to subacromial erosion shape, there were no significant differences in CMS, KSS, VAS score, ROM, and CCD between the groups (Table 2).

**Table 2:** Clinical and radiological outcomes compared between groups divided according to acromion type at final follow-up

	Type I (Simple groove, ***n*** = 13)	Type II (Cave, ***n*** = 2)	Type III (Marginal protrusion, ***n*** = 3)	***P***-value
Functional score				
CMS	96.46 ± 5.55	97.00 ± 4.24	97.67 ± 1.15	0.933
KSS	96.31 ± 6.21	98.00 ± 2.83	99.67 ± 0.58	0.275
VAS	0.92 ± 1.04	1.50 ± 0.71	0.67 ± 0.58	0.335
Range of motion				
Forward elevation	152.31 ± 23.15	170.00 ± 14.14	153.33 ± 20.82	0.467
Abduction	151.54 ± 24.10	170.00 ± 14.14	163.33 ± 5.77	0.318
External rotation at side	58.46 ± 12.14	90.00 ± 0.00	66.67 ± 32.15	0.500
CC distance (%)	10.52 ± 17.23	93.91 ± 125.43	48.01 ± 36.27	0.096

*CMS* Constant-Murley score, *KSS* Korean Shoulder score, *VAS* visual analog score, *CC* coracoclavicular

### Complications

In patients with AC joint dislocations, 4 patients showed reduction loss (subluxation, 2; dislocation, 2; 13%), 1 patients showed shoulder stiffness, and 18 patients showed subacromial erosion, respectively, after implant removal. None of the patients had rotator cuff tears or scapular fractures at before surgery and final follow up.

## Discussion

Many surgeries and different types of devices have been used to treat AC joint dislocations with varying outcomes. One of the surgical techniques that have proven to be effective in the treatment of AC joint dislocations is the use of a clavicle hook plate [7]. Hook plates are widely used as they enable secure fixation against rotational, horizontal, and vertical forces, as well as early motion [28]. Due to posterior displacement of the distal clavicle and severe superior displacement in type 4 and 5 injuries, respectively, most authors have suggested surgical treatment in these cases [3, 8, 20, 23, 29]. Yoon et al. [30] compared the clinical and radiological outcomes of 18 and 24 patients who underwent CC ligament reconstruction and hook plate fixation, respectively, for AC joint dislocation. Both groups achieved satisfactory clinical outcomes; however, maintenance of reduction indicated that hook plate fixation was a better treatment option. Similar to several previous studies, our study showed good clinical results after hook plate fixation in type 5 AC joint dislocations. In addition, radiological results, such as the CCD, at the final follow-up showed good outcomes.

Although the hook plate showed good clinical results, subacromial erosion was observed at a relatively high frequency after surgery [4, 10, 12]. Oh et al. reported that 38% (15/39) of patients treated for AC joint dislocations with hook plate fixation showed subacromial erosion [19]. Lee et al. reported that there were no significant differences in clinical and radiological results between patients with (*n* = 18) and without (*n* = 34) subacromial erosion after AC joint dislocation treatment with hook plate fixation [15]. Of the patients treated for AC joint dislocations (Rockwood type 5) with hook plate fixation in our study, 60% (18/30) showed subacromial erosion. However, both patients with and without subacromial erosions showed good clinical and radiological results. There were no significant differences in clinical results. Thus, although hook plate fixation of an AC joint dislocation can cause subacromial erosion, the resulting erosion does not significantly affect function, including pain.

This is the only study which has investigated whether subacromial erosion shape, evaluated by CT, affects clinical and radiological outcomes in patients treated with hook plate fixation. We hypothesized that if the subacromial erosion has a protruding shape, impingement of the rotator cuff can cause a rotator cuff lesion. Our study showed that there were no significant differences in clinical and radiological results between the groups subdivided by subacromial erosion shape. Moreover, there were no rotator cuff lesions in any of the patients. Our study has some limitations, including its retrospective nature and small sample size. Additionally, long-term follow-up is required to evaluate the effect of the shape of the subacromial erosion on the rotator cuff. Not performing the dynamic US to check the subacromial impingement is also a limitation.

## Conclusions

Whether the shape of the subacromial erosion affects clinical outcomes and complications is unknown. Our study showed that the use of a clavicular hook plate is a good treatment option for AC joint dislocation. Subacromial erosion was a common finding after hook plate fixation in AC joint dislocation. However, the presence or absence of subacromial erosion did not affect functional outcomes at the final follow-up. Additionally, the shape of the subacromial erosion did not affect clinical and radiological results, nor the rotator cuff.

## Data Availability

The datasets used during the present study are available from the corresponding author on reasonable request.

## Abbreviations

AC joint: Acromioclavicular
AP view: Anterior-posterior
CCD: Coracoclavicular distance
PACS: Picture archiving and communication system
CMS: Constant-Murley score
KSS: Korean Shoulder score
ROM: Range of motion
VAS: Visual analog scale
